# Urinary sodium excretion is low prior to acute kidney injury in patients in the intensive care unit

**DOI:** 10.3389/fneph.2022.929743

**Published:** 2022-09-30

**Authors:** David Gomes de Morais, Talita Rojas Cunha Sanches, Mirela Aparecida Rodrigues Santinho, Eduardo Yuki Yada, Gabriela Cardoso Segura, Diogo Lowe, Guilherme Navarro, Victor Faria Seabra, Leandro Utino Taniguchi, Luiz Marcelo Sá Malbouisson, Carmen Diva Saldiva de André, Lúcia Andrade, Camila Eleuterio Rodrigues

**Affiliations:** ^1^ Hospital das Clínicas da Faculdade de Medicina da Universidade de São Paulo (HCFMUSP), Disciplina de Nefrologia, Faculdade de Medicina, Universidade de São Paulo, São Paulo, Brazil; ^2^ Centro de Estatística Aplicada, Instituto de Matemática e Estatística, Universidade de São Paulo, São Paulo, Brazil

**Keywords:** acute kidney injury, intensive care unit, kidney concentrating ability, sodium–hydrogen exchanger 3, kidney tubules, proximal

## Abstract

**Background:**

The incidence of acute kidney injury (AKI) is high in intensive care units (ICUs), and a better understanding of AKI is needed. Early chronic kidney disease is associated with urinary concentration inability and AKI recovery with increased urinary solutes in humans. Whether the inability of the kidneys to concentrate urine and excrete solutes at appropriate levels could occur prior to the diagnosis of AKI is still uncertain, and the associated mechanisms have not been studied.

**Methods:**

In this single-center prospective observational study, high AKI risk in ICU patients was followed up for 7 days or until ICU discharge. They were grouped as “AKI” or “No AKI” according to their AKI status throughout admission. We collected daily urine samples to measure solute concentrations and osmolality. Data were analyzed 1 day before AKI, or from the first to the fifth day of admission in the “No AKI” group. We used logistic regression models to evaluate the influence of the variables on future AKI diagnosis. The expression of kidney transporters in urine was evaluated by Western blotting.

**Results:**

We identified 29 patients as “No AKI” and 23 patients as “AKI,” the latter being mostly low severity AKI. Urinary sodium excretion was lower in “AKI” patients prior to AKI diagnosis, particularly in septic patients. The expression of Na+/H+ exchanger (NHE3), a urinary sodium transporter, was higher in “AKI” patients.

**Conclusions:**

Urinary sodium excretion is low before an AKI episode in ICU patients, and high expressions of proximal tubule sodium transporters might contribute to this.

## Introduction

Acute kidney injury (AKI) is a major global health problem that occurs with high incidence in intensive care unit (ICU) settings (1). It is a disease with high morbidity and mortality rates, and it has been recognized as an important risk factor for chronic kidney disease (CKD) progression and the need for dialysis (2).

An AKI diagnosis is currently based on an elevation in serum levels of creatinine and/or a reduction in urine output (3), parameters that sometimes may lead to delayed diagnosis. Serum creatinine usually presents little or no change until there is kidney damage of up to 50% of nephrons, and reduction in urine output may not occur in some patients. Many efforts have been made by the scientific community to find new biomarkers that could predict AKI or provide a timely diagnosis. Furthermore, many molecules have been successfully identified as potential early markers of AKI presence and severity, such as urine neutrophil gelatinase-associated lipocalin (NGAL), tissue inhibitor of metalloproteinases 2 (TIMP-2), and insulin-like growth factor binding protein 7 (IGFBP-7). The Acute Disease Quality Initiative (ADQI) study group proposed that they should be used as complementary diagnostic criteria for detecting AKI (4). However, most of these new biomarkers remain unavailable and unaffordable to most health services in the world, and their clinical use has been very restricted.

Data from CKD patients show that kidney disease progression may be associated with an augmented loss of free water in the urine and lower urine osmolality (5). In mild to moderate CKD, the inability to concentrate urine causes polyuria and nocturia, which are the initial symptoms of this disease. In AKI patients, urinary sodium concentration decreases as AKI progresses (6), and mortality seems higher in patients experiencing low urine sodium concentration (7). In addition, elevated urinary solute excretion was demonstrated to be a marker of successful weaning from dialysis (8), which suggests that urinary concentration ability may be one of the first steps in kidney recovery after an AKI episode.

Urinary concentration depends on sodium concentration in the outer medulla of the kidney and on sodium and urea concentrations in the inner medulla (9). Therefore, the expression of sodium and urea transporters might be modified in AKI. Under normal conditions, sodium reabsorption occurs at the proximal tubule *via* the luminal membrane Na+/H+ exchanger (NHE3) and the (Na^+^/K^+^)-ATPase located at the basolateral membrane, which is responsible for 60%–70% of overall sodium reabsorption in the kidneys (10). At the thick limb of the loop of Henle, the Na^+^2Cl^-^K^+^ (NKCC2) cotransport may reabsorb up to 20%–30% of total filtered sodium. Active sodium reabsorption at the distal tubule is driven by the Na^+^Cl^-^ (NCC) cotransporter, which is responsible for 5%–10% of overall sodium reabsorption. While at the collecting duct, a small percentage of sodium may be reabsorbed by the collector duct sodium channel (ENaC) (10). Urea content in the medulla of the kidney depends mainly on reabsorption at the collecting duct by UT-A1 and UT-A3 transporters and followed by secretion at the thin limbs of the loop of Henle, mainly *via* UT-A2 in a urea recycling pathway (9).

Whether reduced solute excretion and loss of urine concentration ability are early signals of AKI or function as timely biomarkers in AKI diagnosis remains unanswered. Additionally, the role of kidney transporters in early AKI in humans is yet to be determined.

In this study, we aimed to evaluate if the reduced capacity of the kidneys to concentrate urine and decreased solute excretion could occur prior to AKI diagnosis using the Kidney Disease: Improving Global Outcomes (KDIGO) criteria in patients admitted to the ICU.

## Methods

### Patient selection

This study was a single-center prospective observational study conducted in a large tertiary care hospital in São Paulo, Brazil. We evaluated adult patients who were admitted to three participating ICUs (a medical ICU, a surgical ICU, and a trauma ICU) from December 2017 to December 2019. All patients who were admitted to the ICU and did not have a current diagnosis of AKI were considered for inclusion.

We excluded patients with a baseline estimated glomerular filtration rate <45 ml/min/1.73 m^2^ following the 2009 Chronic Kidney Disease Epidemiology Collaboration (CKD-EPI) formula (11), previous kidney transplantation, limited supportive medical treatment due to advance directives and goals of care, and body mass index <19 kg/m^2^. Patients who were unable to provide urine samples and those not willing to participate in the study were also excluded. We excluded patients who had received an AKI diagnosis in the previous 3 months, whether in the current or previous hospital admission. If a patient was not diagnosed as having AKI, but the serum creatinine concentration decreased more than 0.3mg/dL during the 7-day follow-up period, the patient was excluded as having AKI at admission. Actual inclusion occurred if there were no exclusion criteria present and the patient was considered to have a high AKI risk. A high AKI-risk patient was defined as anyone whose risk prediction clinical score as proposed by Malhotra et al. (12) was ≥5 points.

### Data collection

Selected patients were followed up for 7 days, or less in cases in which death or ICU discharge occurred. If AKI occurred, the follow-up ended on the day of AKI diagnosis. The follow-up consisted of the collection of demographic and clinical data from ICU admission and the daily sampling of urine to evaluate the following biochemical parameters: urea, creatinine, sodium, potassium, and osmolality. Blood tests were performed daily. Serum osmolality was estimated based on serum sodium, urea, and glucose values (serum osmolality = 2 × serum sodium [mmol/l] + serum urea [mg/dl]/6 + serum glucose [mg/dl]/18), and urine osmolality was measured using a vapor pressure osmometer (model 5520; Wescor, Logan, UT, USA). In addition to measuring urine osmolality, we estimated urine osmolarity according to a model proposed by Youhanna et al. (13), in which estimated urine osmolarity = (urinary sodium + urinary potassium) × 2 + urinary urea (all in mmol/l).

### AKI definition

Baseline creatinine was defined as the lowest serum creatinine from 3 months before index hospital admission up to the end of the follow-up period. AKI was defined according to the KDIGO criteria (14), as an increase in serum creatinine by 0.3 mg/dl in 48 h or to 1.5 times baseline creatinine during the 7-day follow-up, or a reduction in urine output to < 0.5 ml/kg/h in 6 h. We retrospectively divided patients into “AKI” and “No AKI” groups.

Patients who developed AKI over the 7-day follow-up period were classified according to the cause of AKI: sepsis, ischemia, or no-sepsis/no-ischemia. We defined any patient presenting with systemic inflammatory response syndrome due to documented or presumed infectious disease as having sepsis-related AKI. Patients who did not fulfill the sepsis criteria and presented with vasoactive drug-dependent hypotension or had undergone major surgeries with a potential of renal ischemia were defined as having ischemia-related AKI. All other “AKI” patients that were not included in the sepsis or ischemia profiles were classified as having no-sepsis/no-ischemia-related AKI. Patients who did not develop AKI but were exposed to the cited causes of AKI were also classified within this groups to better compare them with “AKI” patients.

In patients who developed AKI, urine analysis was performed based on the day before clinical AKI diagnosis (D-1). In patients who did not develop AKI, daily urine parameters from the first 5 days in ICU were considered, and the average among these values was obtained to be compared against “AKI” patients.

### Aims

The primary objective was to determine if urinary concentration and solute excretion could be possible predictors of AKI 1 day before clinical diagnosis. The secondary outcomes were (A) to evaluate the urinary expression of renal transporters in “AKI” and “No AKI” patients; (B) to validate the clinical AKI risk score proposed by Malhotra et al. (12) in our study population; and (C) to evaluate if urinary concentration and solute excretion could be associated with in-hospital mortality.

### Statistical analysis

Statistical analyses were performed with the R statistical software, version 4.0.5, RStudio, version 1.4.1106 (R Development Core Team, 2020), and JMP version 15.2.0 (SAS Institute, Cary, NC, USA).

Continuous variables are described as mean ± standard deviation (SD) or as the median and interquartile range (IQR), as appropriate. Categorical variables are reported as proportions. Comparisons between groups were performed using the compareGroups package in RStudio software (R Development Core Team, 2020) using the appropriate test according to each variable distribution (e.g., t-test, variance analysis, Kruskal-Wallis test, Fisher’s exact test). Differences were considered statistically significant at *p*<0.05.

We used logistic regression models to assess AKI as the outcome. To select variables to include in the adjusted model, a first triage was done including variables with a *p*-value <0.25 in the Kruskal-Wallis test. After this, we performed automated processes to select variables (forward, backward, and stepwise) using stepAIC in R, and variables were selected based on the Akaike information criterion (AIC). Variables related to urinary solutes and concentration and clinically relevant variables were included. We included interaction terms containing the cause of AKI and solute excretion to check if the predictive value of any solute could depend on the cause of AKI. To evaluate the predictive ability of the final model, we constructed a receiver operating characteristic (ROC) curve. The sensitivities and specificities were corrected by applying the “leave-one-out” cross-validation method.

A multiple logistic regression model was also performed to assess in-hospital mortality as the outcome. We included variables related to urinary solute excretion (and we have selected the one with the lowest *p*-value in group comparison) and clinically relevant variables (including known mortality predictors).

To validate the clinical score proposed by Malhotra et al. (12) in our population, we constructed a ROC curve. We included 372 additional patients who were not considered in the main analysis because they had low AKI risk scores (<5) at the initial evaluation.

### Analysis of urinary transporters

In “AKI” patients, we studied urine collected 1 day before AKI diagnosis; in patients who did not develop AKI, pooling of urine samples from the first to the fifth day of ICU admission was done for each patient. We evaluated urinary transporters in patients exposed to sepsis and ischemia. Urine samples (4 ml each) were concentrated with centrifugal filter units (Merck Millipore, Amicon^®^, Darmstadt, Hessen, Germany) to recover proteins bigger than 30 kDa, with a spin time enough to obtain 250-ml samples. The volume of urine applied on each well corresponded to 10 μg of urine creatinine. Samples underwent polyacrylamide gel electrophoresis and then were transferred by electroelution to polyvinylidene fluoride (PVDF) membranes (Hybond-P; GE Healthcare, Buckinghamshire, UK). After a blockade with 5% non-fat dry milk in Tris-buffered saline, the blots were incubated overnight with antibodies against kidney tubule transporter proteins, named proximal Na+/H+ exchanger (NHE3), collector duct sodium channel (ENaC), renal outer medullary potassium channel (ROMK), and aquaporin-2 (AQP2) (Santa Cruz Biotechnology, Santa Cruz, California, USA). The blots were incubated with peroxidase-conjugated secondary antibodies, and visualization was performed with a chemiluminescence detection system (Amersham Biosciences; GE Healthcare; Amersham, UK). Semi-quantitative protein expression was determined by densitometry using Image J software (https://imagej.nih.gov), and values were adjusted to the mean of the “No AKI” group.

### Ethical aspects

The local institutional ethics committee, CAPPesq (Comissão de Ética para Análise de Projetos de Pesquisa do HCFMUSP), approved this study (under the number 71676517.4.0000.0068) and waived informed consent due to the eminently observational nature of the study (under amendment number 2.697.901). All methods used in this study were in accordance with the relevant guidelines and regulations.

## Results

**Figure 1:**
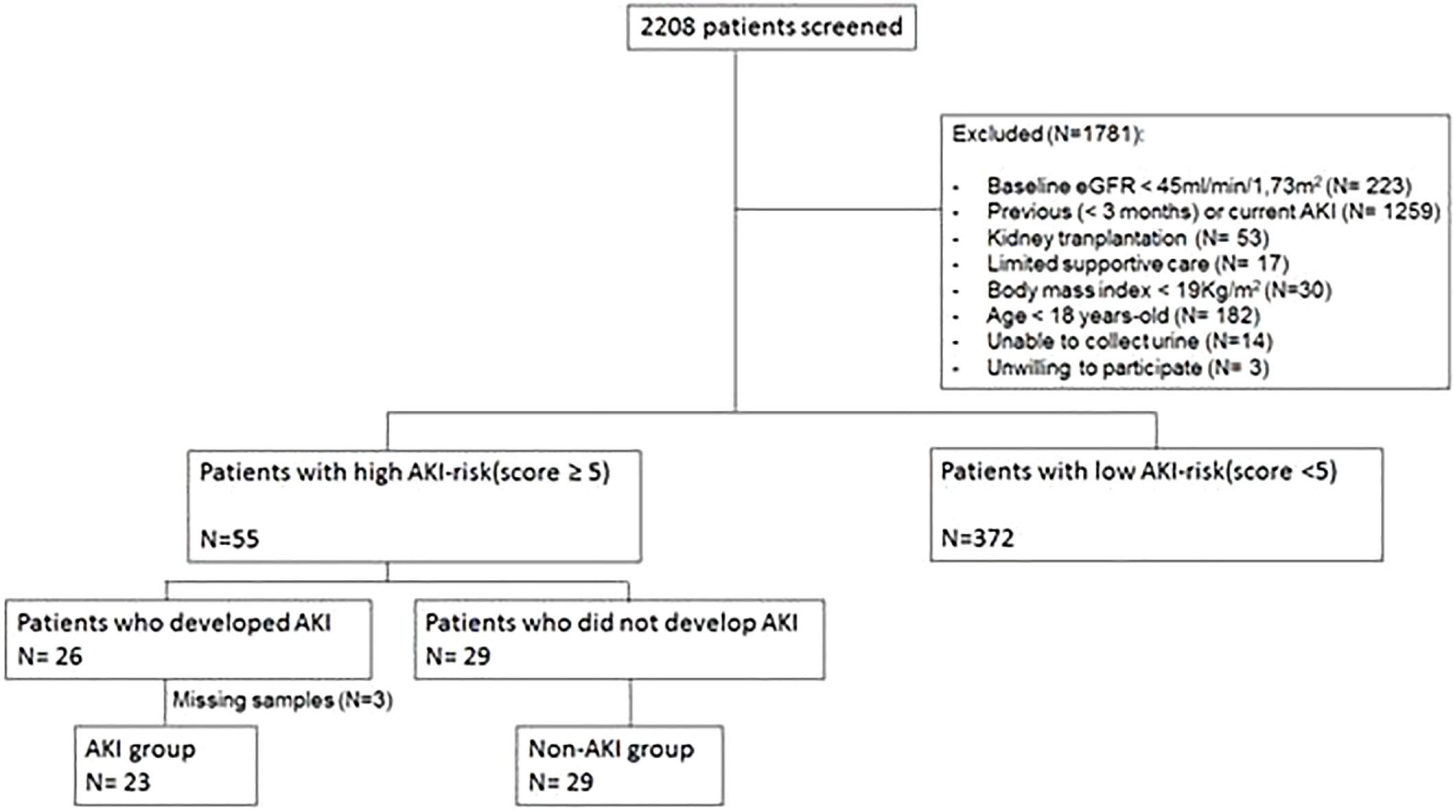
Flowchart of the study population selection. eGFR, estimated glomerular filtration rate; AKI, acute kidney injury.

We included 52 patients who presented with high AKI risk at ICU admission and had urine samples to be analyzed (**Figure 1**). A major exclusion factor was having previous (<3 months) or current AKI (N=1,259). There were 796 patients admitted to the ICU presenting with current AKI and 463 patients who had recent AKI up to 3 months before index admission.

The prevalence of comorbidities was similar between the patients who developed AKI and those who did not. Hypertension was observed in 63.5% of patients, diabetes mellitus in 19.2%, congestive heart failure in 7.7%, cerebrovascular disease in 11.5%, and peripheral vascular disease in 5.8%.

AKI was diagnosed in 26 out of 55 high-AKI risk patients, but urine samples the day before AKI diagnosis could not be collected in three patients. Thus, there are 23 patients in the “AKI” group analysis. In the high-AKI risk group, 29 patients did not develop AKI, and they are identified as the “No AKI” group (**Figure 1**).

In the “AKI” group, 13 patients developed stage 1 (57%) AKI. Stage 2 AKI occurred in seven patients (30%), and stage 3 AKI occurred in three patients (13%). Attributable causes of AKI were considered to be sepsis in 8 out of 23 patients, low perfusion in 5 patients, and no-sepsis/no-ischemia in 10 patients. Most patients in the no-sepsis/no-ischemia group were exposed to iodine contrast (8 out of 10 patients).

**Table 1** shows the main characteristics of the patients and provides urine and serum biochemical analysis within each group by AKI status. “AKI” patients had higher acute disease severity scores (simplified acute physiology score, SAPS3; and sequential organ failure assessment, SOFA score) and higher serum urea and creatinine levels than the “No AKI” group, even 1 day before AKI diagnosis. Urinary urea and urine osmolality were not different between groups, but urinary sodium excretion was lower in the “AKI” group when compared to the “No AKI” group. “AKI” patients exposed to sepsis seemed to present with particularly low urinary sodium excretion, but this did not occur in the “No AKI” group (**Supplementary Table 1**).

**Table 1:** Characteristics of the patients by acute kidney injury status.

	‘AKI’ (N=23)	‘No AKI’ (N=29)
Age, years, median [IQR] ^&^	57.0 [45.5; 67.0]	58 .0 [47.0; 65.0]
Male sex, n (%)	12 (52.2)	9 (31.0)
White race, n (%)	19 (82.6)	21 (72.4)
Body mass index, Kg/m^2^, median [IQR] ^&^	23.4 [21.1; 27.7]	23.4 [21.5; 25.4]
SAPS3 score, mean ± SD ^&^ *	55.3 ± 16.7	46.0 ± 14.9
SOFA score, median [IQR] ^#^ *	4.0 [2.5; 6.5]	1.0 [0.0; 2.0]
Non-renal SOFA score, median [IQR] ^#^ *	4.0 [2.5; 6.5]	1.0 [0.0; 2.3]
AKI risk prediction clinical score (12), median [IQR] ^&^	7 [5-8]	5 [5-7]
Norepinephrine dose, μg/kg/min, median [IQR] ^&^	0.04 [0.00; 0.14]	0.00 [0.00; 0.08]
Mechanical ventilation, n (%) ^&^	18 (78.3)	16 (55.2)
Baseline creatinine, mg/dl, median [IQR]	0.57 [0.53; 0.78]	0.56 [0.43; 0.66]
Urine output, ml/kg/h, median [IQR] ^#^	0.80 [0.70; 1.30]	0.90 [0.70; 1.20]
Fluid balance, ml/24h, median [IQR] ^#^	230 [-198; 736]	111 [-495; 675]
Serum creatinine, mg/dl, median [IQR] ^#^ *	0.69 [0.61; 0.93]	0.59 [0.52; 0.75]
Serum urea, mg/dl, median [IQR] ^#^ *	37.0 [24.0; 44.0]	21.0 [18.0; 28.0]
Serum sodium, mEq/l, median [IQR] ^#^	143 [139; 146]	141 [139; 143]
Serum potassium, mEq/l, mean ± SD ^#^	3.92 ± 0.54	3.86 ± 0.36
Serum osmolality, mOsm/Kg, mean ± SD ^#^	297 .0 ± 14.4	295.0 ± 11.9
Serum urea / serum creatinine ratio, median [IQR] ^#^	42.3 [33.3; 61.9]	34.7 [29.4; 51.8]
Urinary creatinine, g/l, median [IQR] ^#^	0.90 [0.80; 1.50]	1.10 [0.70; 1.50]
Urinary urea, g/l, median [IQR] ^#^	13.5 [8.1; 18.4]	12.4 [9.9;15.7]
Urinary sodium, mEq/l, median [IQR] ^#^	59.5 [23.0; 109.8]	90.0 [52.5; 146.7]
Urinary potassium, mEq/L, mean ± SD ^#^	56.7 ± 30.7	47.9 ± 23.3
Measured urinary osmolality, mOsm/Kg H2O, mean ± SD #	593.5 ± 261.3	611.4 ± 237.7
Estimated urinary osmolarity, mmol/l, mean ± SD ^#^	494 .0 ± 177.3	540.7 ± 185.0
Urinary urea excretion, g/24h, median [IQR] #,	18.0 [9.6; 29.8]	17.5 [13.1; 24.4]
Fractional excretion of urea, %, mean ± SD ^#^	30.4 ± 14.1	34.0 ± 11.1
Urinary urea / urinary creatinine ratio, median [IQR] ^#^	13.2 [8.4;20.4]	11.4 [10.6;15.8]
Urinary sodium excretion, mEq/24h, median [IQR] ^#^	103.0 [34.6; 135.5]	126.3 [52.7; 286.4]
Fractional excretion of sodium, %, median [IQR] ^#^	0.24 [0.14; 0.56]	0.39 [0.23; 0.86]
Urinary sodium / urinary creatinine ratio, median [IQR] ^#^*	55.1 [20.6; 121.8]	95.8 [52.0; 174.4]
Fractional excretion of potassium, %, median [IQR] ^#^	9.5 [6.8; 11.7]	7.0 [5.0; 9.2]
Urinary sodium+urinary potassium, mEq/l, median [IQR] ^#^	133.0 [98.4; 172.0]	137.0 [108.0; 187.0]
Diuretic use, n (%)	1 (4.4)	2 (6.9)
Vasopressin use, n (%)	1 (4.4)	1 (3.5)
RAAS inhibitors use, n (%)	3 (13.0)	4 (14.3)

^#^Data from the “AKI” group: the day before AKI diagnosis. Data from the “No AKI” group: average of the first 5 days for each variable. ^&^Data from the first day of ICU admission. ^*^p<0.05 between groups. AKI, acute kidney injury; IQR, interquartile range; n, number of patients; RAAS, renin-angiotensin-aldosterone system; SAPS, simplified acute physiology score; SD, standard deviation; and SOFA, sequential organ failure assessment.

**Figure 2:**
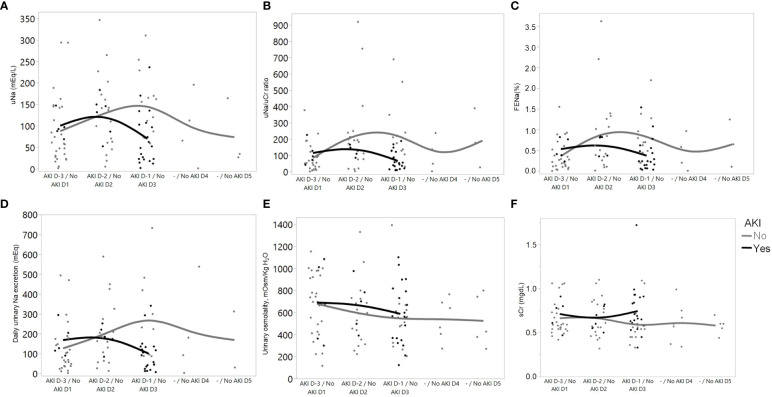
Trajectories of variables over time in patients who developed AKI (from 3 days before AKI diagnosis (D-3) to 1 day before AKI diagnosis (D-1)), and with “No AKI” (over the first 5 days of ICU admission). Data from D-4 were not included in the graph because of the low number of patients (n=2). **(A)** Urinary sodium concentration; **(B)** Urinary sodium/urinary creatinine (uNa/uCr) ratio; **(C)** Fractional excretion of sodium; **(D)** Daily urinary sodium excretion; **(E)** Measured urinary osmolality; and **(F)** Serum creatinine.

**Figure 2** depicts the trajectories of urinary sodium excretion, urinary osmolality, and serum creatinine over the course of the follow-up period in patients from both groups. Despite having no change in urinary osmolality, patients with AKI presented a reduction in urinary sodium excretion immediately before AKI occurrence.

Only two patients in the “No AKI” group and one patient in the “AKI” group received diuretic drugs during the study, precluding any meaningful analysis regarding interference of diuretics in urinary sodium excretion. Similarly, only three patients in the “No AKI” group and two patients in the “AKI” group received fluids upon ICU admission, so this variable was not included as a covariant in any model studied here.

In-hospital mortality was 34.8% in the “AKI” group and 13.8% in the “No AKI” group (p=0.10).

### Influence of variables on future AKI diagnosis

Urinary osmolality and solute excretion-related variables were evaluated according to their ability to predict AKI in simple logistic regression models (**Supplementary Table 2**). A low urinary sodium/urinary creatinine ratio was related to future AKI when evaluated 1 day prior to AKI diagnosis. The cause of AKI was not indicated as possibly interfering in solute excretion.

Other factors known to predict AKI, such as acute severity of disease (evaluated here by non-renal SOFA) and baseline creatinine, were also assessed. Non-renal SOFA 1 day before AKI diagnosis was the best predictor for AKI on the following day (OR 95% CI: 1.79 [1.33; 2.66], *p*<0.01, c-statistic: 0.84 at simple logistic regression model), while baseline creatinine was not considered a predictor in the studied population (p=0.13).

The multivariable logistic regression models for predicting AKI (urinary sodium/urinary creatinine ratio, non-renal SOFA score, and baseline serum creatinine) are shown in **Table 2**. The complete model (Model 4) was considered the best predictor model, with the lowest Akaike information criterion and highest c-statistic. After the leave-one-out cross-validation, Model 4 still presented a high c-statistic value: 0.828 (95% CI: 0.696–0.940) (**Supplementary Figure 1**). Sensitivity and specificity values for AKI prediction after adjustment were 78.3% and 72.4%, respectively.

**Table 2:** Multiple logistic regression models, with acute kidney injury as the outcome.

	Model 1 (c-stat: 0.67)		Model 2 (c-stat: 0.70)		Model 3 (c-stat: 0.86)		Model 4 (c-stat: 0.87)		Model 5 (c-stat: 0.85)	
	AIC: 69.38		AIC: 68.61		AIC: 55.18		AIC: 54.09		AIC: 55.71	
	OR (95% CI)	*p*-value	OR (95% CI)	*p*-value	OR (95% CI)	*p*-value	OR (95% CI)	*p*-value	OR (95% CI)	*p*-value
**uNa/uCr**	0.99 (0.98; 0.998)	0.046	0.99 (0.98; 0.998)	0.05	0.99 (0.98; 1.00)	0.10	0.99 (0.98; 1.00)	0.10		
Non-renal SOFA*					1.74 (1.29; 2.58)	<0.01	1.77 (1.30; 2.68)	<0.01	1.81 (1.34; 2.71)	<0.01
**Baseline sCr**			7.88 (0.70; 128.79)	0.11			11.77 (0.76; 310.63)	0.10	11.54 (0.79; 309.96)	0.10

AIC, Akaike information criterion; CI, confidence interval; c-stat, c-statistic; OR, odds ratio; SOFA, sequential organ failure assessment; sCr, serum creatinine; and uNa/uCr, urinary sodium/urinary creatinine ratio.

### Clinical score validation

To validate the clinical score in Malhotra et al. (12) that was used as screening for AKI high risk in our study, we performed an exploratory analysis with 427 patients (55 patients with high AKI-risk score ≥5 and 372 patients with low AKI-risk score <5) (**Supplementary Figure 2**). The ROC area under the curve (AUC) reflected that the clinical score in our population was 0.69 (95% CI: 0.61–0.77). The 5-point threshold was the one with the best performance regarding specificity (92.1%) and sensitivity (44.1%), with positive and negative predictive values of 47.3% and 91.1%, respectively (**Supplementary Figure 3**).

### Expression of urinary transporters

We analyzed the urinary expression of the renal transporters Na+/H+ exchanger (NHE3), collector duct sodium channel (ENaC), renal outer medullary potassium channel (ROMK), and aquaporin-2 (AQP2) in patients exposed to sepsis and ischemia.

**Figure 3:**
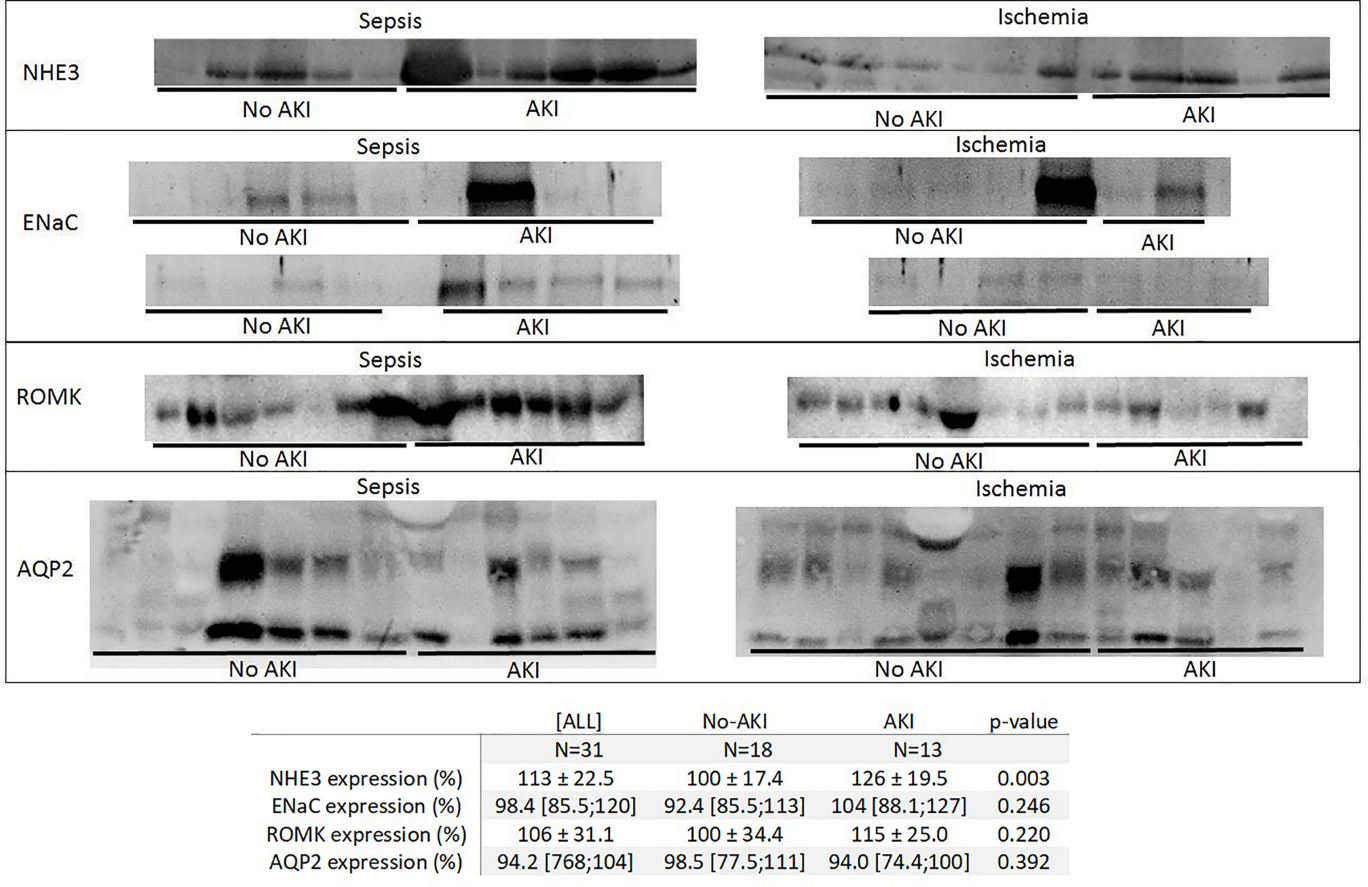
Immunoblotting of the Na+/H+ exchanger (NHE3), collector duct sodium channel (ENaC), renal outer medullary potassium channel (ROMK), and aquaporin-2 (AQP2) expression in urine in “No AKI” and “AKI” patients, by exposure to sepsis or ischemia. In “AKI” patients, urine was collected 1 day before AKI diagnosis; in “No AKI” patients, pooled urine samples were collected from each patient from the first to the fifth day of ICU admission. Each continuous strip represents a different blot, and each blot contains samples from the “AKI” and “No AKI” patients to help with the visual comparison between the groups.

“AKI” patients had higher NHE3 expression than “No AKI” patients (126 ± 19.5% vs. 100.0 ± 17.4%, *p*=0.003). ENaC, ROMK, and AQP2 expressions were not different between “AKI” and “No AKI” (**Figure 3**). **Supplementary Table 3** presents the densitometric analysis of these transporters in the four subgroups: “No AKI and sepsis,” “AKI and sepsis,” “No AKI and ischemia,” and “AKI and ischemia.” Although the low number of patients per subgroup precludes any meaningful comparison among them, it seems that NHE3 expression might be higher before AKI in both sepsis and ischemia patients. However, ENaC and ROMK expressions seem to be somehow increased only in patients with sepsis and AKI.

### Differences in urinary solute excretion by in-hospital mortality

Survivors and non-survivors differed in many clinical variables in this study. **Supplementary Table 4** shows the main characteristics of patients according to in-hospital survival. Aside from differences in scores measuring disease severity (i.e., SAPS3, SOFA, non-renal SOFA, and the AKI risk prediction clinical score in Malhotra et al.), non-survivors exhibited higher serum urea and serum sodium and lower urinary solute excretion. A multiple logistic regression model was constructed to examine which variables could better predict in-hospital mortality, and the results are shown in **Supplementary Table 5**. There was no correlation between serum sodium, and urinary sodium and potassium excretion (Spearman r 0.10, *p*=0.47).

High SOFA score, age, and serum sodium were independent risk factors for in-hospital mortality. Low urinary excretion of sodium and potassium added a predictive value in the model, as removal of this variable would lead to higher AIC (36.7) and lower c-statistic (0.95).

## Discussion

This study approaches some differences between urine solute excretion and the expression of urinary renal transporters 1 day before AKI diagnosis in patients who developed AKI in ICU and those patients without AKI during their admission. We aimed to determine if urinary concentration and solute excretion could identify patients who would develop AKI 1 day before clinical diagnosis.

Our data showed that urinary osmolality was equal in the “AKI” and “No AKI” groups. Despite this, urinary sodium excretion was lower in the “AKI” group. A low urinary sodium/creatinine ratio was considered a contributing risk to AKI development on the following day in the univariable analysis, and even after an adjustment to baseline serum creatinine, this variable seemed to be important in AKI prediction. After an adjustment to the acute severity of the disease, however, it added little information to the non-renal SOFA score as a predictor of AKI.

In the past, patients who presented with AKI and low urinary sodium excretion were considered to have renal hypoperfusion, and fluid infusion was sometimes proposed in this scenario. This paradigm has already been modified in recent years, and decreasing sodium excretion has been shown to occur regardless of low renal blood flow, particularly in sepsis-related AKI (15). Contrary to prior belief, sodium urinary excretion has now been demonstrated to not be a good tool for differentiating transient from persistent AKI (16).

However, it has been previously demonstrated that urinary sodium excretion can be reduced in mild to moderate initial AKI, and studies have already shown that low urinary sodium excretion may occur prior to the clinical diagnosis of AKI. Acute variations in urinary sodium excretion have been proposed to be a consequence of acute derangements in the glomerular filtration rate (17). In this context, sodium excretion would be reduced regardless of the cause of AKI. Nevertheless, sodium urinary excretion may be a dual-phase process as AKI progresses. While there is an initial decrease in urinary sodium excretion at the inception of AKI, as long as the disease becomes more severe, an impairment in tubular sodium reabsorption occurs, and urinary sodium excretion may increase (6, 17, 18).

The expression of urinary renal transporters was consistent with biochemical data, as demonstrated by higher sodium reabsorption channels being found in “AKI” patients, which is consistent with the lower sodium excretion in these patients. We could not demonstrate any difference in aquaporin-2 expression in “AKI” patients, and this could partially explain why AKI was not associated with lower urine osmolality. We hypothesize that other solutes not evaluated in this study may be excreted more in urine to compensate for the lower sodium excretion. Thus, it seems that, at least in mild AKI, urinary sodium excretion, more than urine concentration inability, is a feature found early in the AKI course.

Both proximal and distal tubular cell dysfunction may occur in AKI (15), and the cause of the damage may have an impact on the distribution of injured cells. In ischemic and toxic AKI, the outer medullar proximal tubule (particularly the S3 segment) is recognized as the most affected segment because there is persistent reduced blood flow in this part of the kidney (19, 20). In sepsis, S2 and S3 segments have already been underscored as pivotal injured sites along the nephron (21). However, sepsis-related AKI is frequently accompanied by heterogeneous distribution of blood flow caused by intense microcirculatory dysfunction (22), and patchy tubular injury may occur in this AKI etiology. NHE3 transporter, located in proximal tubules, seemed affected by AKI in both ischemia and sepsis. However, more distal transporters, such as ENaC and ROMK, appeared somehow affected mainly in sepsis but not in ischemia.

In animal sepsis models, the opposite was previously described: Higher urinary sodium excretion and lower expression of sodium reabsorption transporters in the kidneys of rats submitted to cecal ligation and puncture (23). A decrease in tubular sodium transporters seems to be associated with high tissue cytokine concentration (24). Lipopolysaccharide injection, cecal ligation, and puncture models have been shown to correlate with reduced expression of NHE3, NKCC2, NCC, ENaC, sodium, potassium adenosine triphosphatase (N+/K+ATPase), and AQP2 (24–26). Unilateral renal arterial obstruction leading to hypoperfusion does not change the gene expression of tubular sodium transporters, but ischemia-reperfusion injury, which is already known to be associated with renal inflammation, decreases renal sodium transporters as well (24). The experimental models that achieved these findings, however, lead to very severe AKI, which is different from what we found in our population.

Thus, we might speculate that in less severe initial renal damage, low urinary sodium excretion may be a marker of AKI to come, with higher expression of sodium reabsorption transporters. However, in more severe cases, the opposite may occur if tubular transporters are reduced. When tubular cells are still intact or mildly damaged, reabsorption of sodium may be possible. However, more severe injury may result in tubule cells being unable to reabsorb solutes. Urine chemistries may be dependent on the severity and phase of AKI during which time they are obtained (27).

Patients who developed AKI after ICU admission usually presented with a mild KDIGO stage 1 AKI. It is possible that most patients who would develop AKI had already presented with this diagnosis before being admitted to the ICU and were then excluded from our study. Our results, in this case, may reflect the condition of a large and busy tertiary hospital in which patients with less severe diseases are not likely to be placed in an ICU and may be placed under critical care often only when they are already under multi-organ dysfunction. Additionally, other authors have already shown that most patients who develop AKI initially presented with this diagnosis outside of an ICU setting (28). We only studied patients in ICU.

However, even if high urinary sodium excretion and the expression of low sodium transporters seem worse than the opposite, having lower than expected sodium and potassium also does not seem to be good. Urinary cations (Na+K) excretion was lower in patients who died in the hospital and added information to the model developed to check association of clinical variables with in-hospital mortality.

In otherwise healthy populations, urinary sodium and potassium excretion have been used as a surrogate for intake (29). The association between urinary sodium excretion and cardiovascular events is J-shaped in these groups: High urinary sodium excretion is considered a risk factor for cardiovascular events, while low urinary sodium excretion is associated with higher cardiovascular risk, death, and hospitalization due to heart failure (30). Low potassium urinary excretion is also considered a risk for death and cardiovascular events (29, 30). In this study, non-survivors presented with a lower urinary sodium/creatinine ratio, lower sodium and potassium urinary excretion, and higher serum sodium when compared to the survivors.

Hypernatremia has already been recognized as an important mortality risk factor in critically ill patients (31–33), and urine cation (Na + K) excretion has been previously demonstrated to be better associated with hypernatremia in intensive care patients than sodium intake or fluid balance (34). Thus, it seems that impaired renal solute excretion might represent a possible marker of severity in critically ill patients.

One of the secondary objectives of this study was to validate in our population the clinical AKI score developed by Malhotra et al. (12). In the original study, areas under ROC curves to predict AKI after ICU admission were 0.79 (95% CI: 0.70–0.89) in the generation cohort and 0.81 (95% CI: 0.78–0.83) in the validation cohort. Positive predictive values for the 5-point cutoff varied between 22.7% and 31.8%, and negative predictive values varied between 96.1% and 95.4%, depending on whether the analysis was performed in the test or validation cohort. In this study, we found that ROC AUC in our population was 0.69 (95% CI: 0.61–0.77), and the same 5-point threshold was indicated as optimal in relation to both specificity and sensitivity. In this study, positive and negative predictive values and specificity and sensitivity were 47.3%, 91.1%, 92.1%, and 44.1%, respectively. Despite having lower ROC curve AUCs, our numbers are not so different from the ones generated by the original study. Thus, as these results seem comparable to our results, more information has been added to generalise its use in the broader population.

We recognize that our study has many limitations. It is a unicentric observation of a few patients with low severity AKI, making our findings difficult to generalize. However, it gives us some insights into the pathophysiology of AKI and might suggest some practical measures, such as using the clinical score to assess AKI risk and implementing sequential measurements of urinary solutes to the list of tests to be done in patients in ICU. As a simple, innocuous, and affordable test, it seems to be a useful supplemental tool in our kidney care arsenal.

## Conclusions

Low excretion of urinary sodium is seen in patients who develop AKI in ICU 1 day prior to AKI diagnosis, and impaired renal sodium and potassium excretion might represent a possible marker of severity in critically ill patients. Higher concentrations of sodium urinary transporters may be the corresponding pathophysiological marker of these findings.

## Data availability statement

The data sets including accession number(s) (Urinary Solute Excretion in ICU) for this study can be found in ZENODO (https://zenodo.org/record/6496521#.Ymi7itpBzIU).

## Ethics statement

This study involving human participants was reviewed and approved by CAPPesq (Comissão de Ética para Análise de Projetos de Pesquisa do HCFMUSP), under the number 71676517.4.0000.0068. The ethics committee waived the requirement of written informed consent due to the eminently observational nature of the study (under amendment number 2.697.901). All methods used in this study were in accordance with the relevant guidelines and regulations.

## Author contributions

DM, GS, and CR acquired the data; MS, TS, and DM performed laboratory analysis and western blot experiments; EY, DL, GN, CA, and CR were responsible for data analysis and interpretation; VS, LT, LM, and LA contributed greatly to the design of the work; CR conceived the study idea and design and wrote the paper. All authors read the final manuscript and approved this final version.

## Funding

This study was funded by the São Paulo State Research Support Foundation (FAPESP - Fundaç̧ão de Amparo à Pesquisa do Estado de São Paulo), grant number: 2016/21301-7, and by the University of São Paulo School of Medicine Foundation, CG 19108.

## Acknowledgments

Some of these data were accepted as an abstract for oral presentation at the Annual International Conference: AKI & CRRT 2019. It was presented on 26 February 2019 in San Diego, CA, USA.

## Conflict of interest

The authors declare that the research was conducted in the absence of any commercial or financial relationships that could be construed as a potential conflict of interest.

The reviewer (GM) declared a shared affiliation to the handling editor, without collaboration with the authors at the time of review.

## Publisher’s note

All claims expressed in this article are solely those of the authors and do not necessarily represent those of their affiliated organizations, or those of the publisher, the editors and the reviewers. Any product that may be evaluated in this article, or claim that may be made by its manufacturer, is not guaranteed or endorsed by the publisher.
