# Supplementary material for: Urinary sodium excretion is low prior to acute kidney injury in patients in the intensive care unit
Source: Front Nephrol. 2022 Sep 30;2:929743. doi: 10.3389/fneph.2022.929743 (PMC10479577; doi:10.3389/fneph.2022.929743)
Supplement: Supplementary file 1 [file DataSheet_1.docx]

Supplementary Material

Supplemental Figure 1: ROC curve for Model 4 (non-renal SOFA score, baseline serum creatinine and urinary sodium / urinary creatinine ratio) as AKI predictor, adjusted by “leave-one-out” cross-validation method. C-statistic = 0.828 (95% CI = 0.696 - 0.940).

**
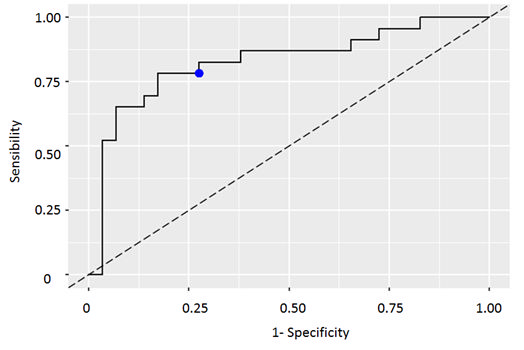
**

Supplemental Figure 2: Malhotra^12^ AKI risk prediction clinical score according to AKI status in this study population.


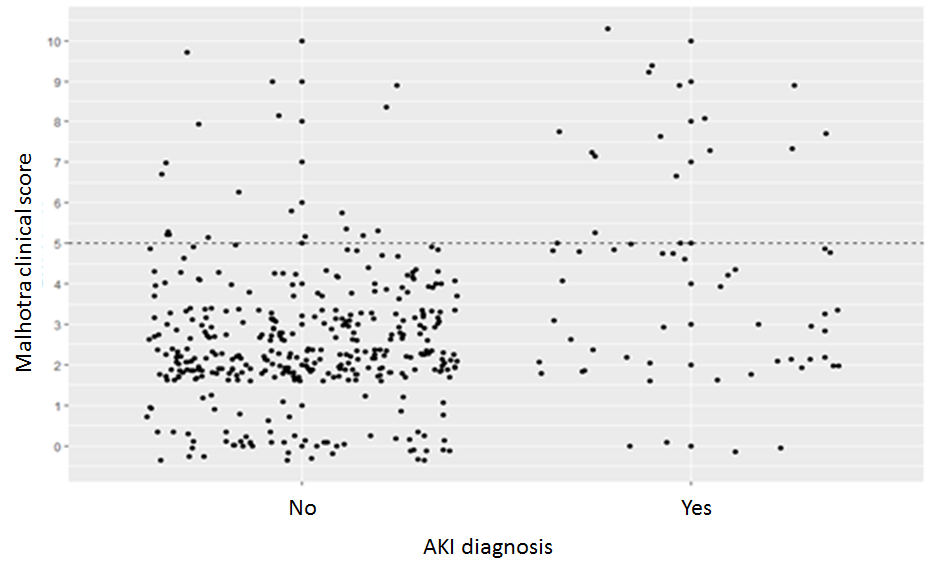


Supplemental Figure 3: ROC curve showing the performance of Malhotra^12^ clinical score in predicting AKI in this study population.


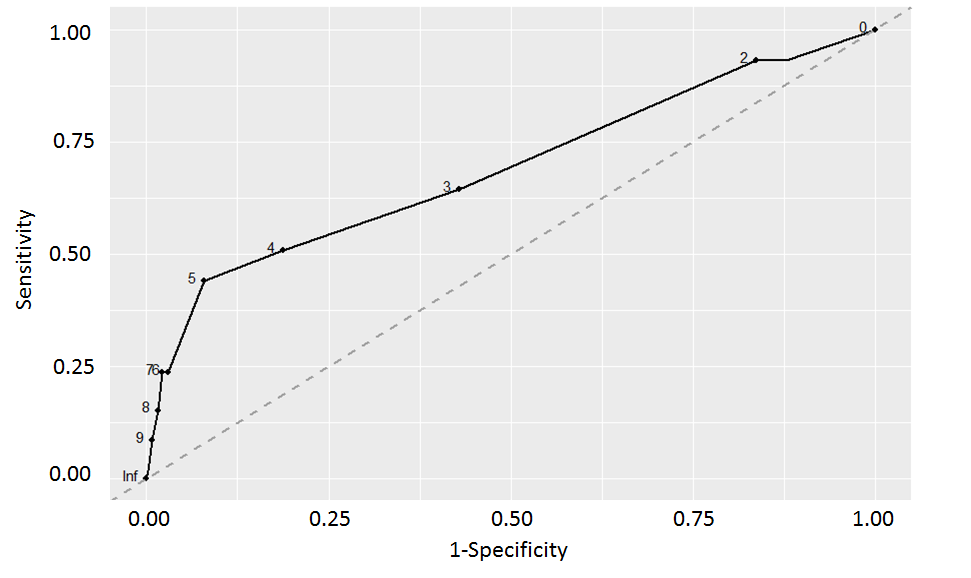


Supplemental Table 1: Solute urinary excretion in groups AKI and No-AKI, by exposure to ischemia, sepsis or no-sepsis/no-ischemia.

| **AKI group (N=23)** | **Ischemia (N=5)** | **Sepsis (N=8)** | **No-sepsis/No-ischemia (N=10)** |
| --- | --- | --- | --- |
| Urinary creatinine, g/l, median [IQR] ^#^ | 0.80 [0.80;0.80] | 1.25 [0.74;2.02] | 1.06 [0.81;1.37] |
| Urinary urea, g/l, mean ± SD ^#^ | 11.6 ± 4.33 | 16.2 ± 6.17 | 13.2 ± 8.26 |
| Urinary sodium, mEq/l, median [IQR] ^#^ | 73.8 [48.6;96.8] | 28.8 [21.0;72.1] | 82.8 [33.5;144] |
| Urinary potassium, mEq/L, mean ± SD ^#^ | 33.6 ± 13.2 | 69.7 ± 28.2 | 57.9 ± 34.0 |
| Measured urinary osmolality, mOsm/Kg H_2_O, mean ± SD ^#^ | 440 ± 154 | 598 ± 210 | 667 ± 321 |
| Estimated urinary osmolarity, mmol/l, mean ± SD ^#^ | 395 ± 109 | 514 ± 173 | 527 ± 203 |
| Urinary urea excretion, g/24h, median [IQR] ^#^ | 11.3 [9.60;31.0] | 19.0 [14.4;23.1] | 16.6 [8.25;29.6] |
| Fractional excretion of urea, %, mean ± SD ^#^ | 31.1 ± 11.6 | 23.9 ± 11.2 | 35.2 ± 16.2 |
| Urinary urea / urinary creatinine ratio, median [IQR] ^#^ | 12.5 [10.2;15.9] | 13.9 [8.40;18.9] | 11.2 [7.05;21.8] |
| Urinary sodium excretion, mEq/24h, median [IQR] ^#^ | 120 [117;133] | 44.5 [30.3;76.6] | 104 [55.5;170] |
| Fractional excretion of sodium, %, median [IQR] ^#^ | 0.48 [0.16;0.62] | 0.23 [0.07;0.31] | 0.34 [0.17;0.72] |
| **No AKI group (N=29)** | **Ischemia (N=10)** | **Sepsis (N=9)** | **No-sepsis/No-ischemia (N=10)** |
|  |  |  |  |
| Urinary sodium / urinary creatinine ratio, median [IQR] ^#^ | 87.6 [39.9;122] | 39.1 [15.9;55.2] | 65.2 [40.1;163] |
| Fractional excretion of potassium, %, median [IQR] ^#^ | 8.34 ± 2.70 | 11.3 ± 6.70 | 10.2 ± 5.10 |
| Urinary sodium + urinary potassium, mEq/l, median [IQR] ^#^ | 101 ± 43.4 | 122 ± 46.5 | 154 ± 88.2 |
| Urinary creatinine, g/l, median [IQR] ^#^ | 1.15 [0.92;2.00] | 0.70 [0.50;1.10] | 1.20 [1.10;1.50] |
| Urinary urea, g/l, median [IQR] ^#^ | 13.1 [10.6;16.0] | 10.4 [8.70;11.9] | 12.7 [11.3;17.0] |
| Urinary sodium, mEq/l, median [IQR] ^#^ | 86.8 [52.8;105] | 122 [69.0;145] | 95.7 [61.8;184] |
| Urinary potassium, mEq/L, mean ± SD ^#^ | 48.6 ± 12.7 | 52.0 ± 33.2 | 43.6 ± 22.5 |
| Measured urinary osmolality, mOsm/Kg H_2_O, median [IQR] ^#^ | 664 [464;765] | 574 [364;600] | 496 [428;866] |
| Estimated urinary osmolarity, mmol/l, median [IQR] ^#^ | 527 [423;596] | 490 [442;572] | 498 [424;736] |
| Urinary urea excretion, g/24h, mean ± SD ^#^ | 18.2 ± 7.99 | 17.7 ± 8.95 | 18.6 ± 6.19 |
| Fractional excretion of urea, %, mean ± SD ^#^ | 30.9 ± 9.36 | 39.4 ± 14.2 | 32.4 ± 8.45 |
| Urinary urea / urinary creatinine ratio, median [IQR] ^#^ | 11.4 [10.3;12.5] | 11.9 [11.2;19.3] | 11.1 [10.6;14.6] |
| **No AKI group (N=29)** | **Ischemia (N=10)** | **Sepsis (N=9)** | **No-sepsis/No-ischemia (N=10)** |
|  |  |  |  |
| Urinary sodium excretion, mEq/24h, median [IQR] ^#^ | 114 [60.5;155] | 127 [67.7;325] | 116 [64.4;255] |
| Fractional excretion of sodium, %, median [IQR] ^#^ | 0.32 [0.26;0.60] | 0.55 [0.18;0.83] | 0.40 [0.29;0.80] |
| Urinary sodium / urinary creatinine ratio, median [IQR] ^#^ | 82.2 [50.8;141] | 191 [50.6;287] | 101 [66.0;116] |
| Fractional excretion of potassium, %, median [IQR] ^#^ | 6.14 [4.99;8.23] | 9.23 [7.83;11.3] | 6.35 [4.72;7.36] |
| Urinary sodium + urinary potassium, mEq/l, mean ± SD ^#^ | 146 ± 74.0 | 157 ± 61.3 | 165 ± 89.8 |

^#^ Data from AKI group regards the day before AKI diagnosis. Data from ‘No AKI’ group regards the first five days average. P=NS

Supplemental Table 2: Simple logistic regression models, AKI as the outcome.

|  | OR (95% CI) | p-value | c-statistic |
| --- | --- | --- | --- |
| uNa (mEq/l) | 0.99 (0.98; 1.00) | 0.08 | 0.65 |
| UVNa 24h (mEq) | 0.99 (0.99; 1.00) | 0.06 | 0.63 |
| FENa (%) | 0.57 (0.15; 1.87) | 0.37 | 0.61 |
| uNa/uCr | 0.99 (0.98; 0.998) | 0.046 | 0.67 |
| uU (g/l) | 1.00 (0.92; 1.09) | 0.91 | 0.50 |
| UVU 24h (g) | 1.02 (0.97; 1.08) | 0.34 | 0.51 |
| FEU (%) | 0.98 (0.93; 1.02) | 0.29 | 0.59 |
| uU/uCr | 1.03 (0.95; 1.12) | 0.50 | 0.52 |
| uK (mEq/L) | 1.01 (0.99; 1.03) | 0.24 | 0.60 |
| FEK, %, | 1.14 (1.00; 1.32) | 0.058 | 0.66 |
| Measured urinary osmolality (mOsm/Kg H_2_O) | 1.00 (0.997; 1.002) | 0.79 | 0.52 |
| Estimated urinary osmolarity (mmol/l) | 1.00 (0.995; 1.002) | 0.36 | 0.56 |

CI: confidence interval; FEK: fractional excretion of potassium; FENa: fractional excretion of sodium; FEU: fractional excretion of urea; OR: odds ratio; uK: urinary potassium concentration; uNa: urinary sodium concentration; uNa/uCr: urinary sodium / urinary creatinine ratio; uU: urinary urea concentration; uU/uCr: urinary urea / urinary creatinine ratio; UVNa 24h: urinary sodium excretion in 24h; UVU 24h: urinary urea excretion in 24h

^#^ Data from ‘AKI’ group regards the day before AKI diagnosis. Data from ‘No AKI’ group regards the first five days average for each variable.

Estimated urinary osmolarity was done as (uNa+uK) X 2 + uU, where uNa is urinary sodium, uK is urinary potassium and uU is urinary urea (solutes in mmol/l) ^13^

Supplemental Table 3: Densitometric analysis of renal transporters in urine according to AKI status and exposure to sepsis or ischemia.

|  | [ALL] | No-AKI and sepsis | AKI and sepsis | No-AKI and ischemia | AKI and ischemia | p.overall |
| --- | --- | --- | --- | --- | --- | --- |
|  | N=31 | N=9 | N=8 | N=9 | N=5 |  |
| NHE3 expression (%) | 113 (22.5) | 100.0 (19.8) | 129 (18.5) | 100 (17.0) | 123 (22.4) | 0.037 |
| ENaC expression (%) | 98.4 [85.5;120] | 93.8 [85.5;119] | 126 [86.3;166] | 91.6 [83.2;110] | 101 [98.4;104] | 0.590 |
| ROMK expression (%) | 106 (31.1) | 100.0 (32.8) | 131 (16.8) | 100.0 (38.0) | 95.0 (18.0) | 0.165 |
| AQP2 expression (%) | 94.2 [76.8;104] | 93.2 [71.9;120] | 94.2 [81.3;97.9] | 99.6 [81.0;106] | 92.9 [71.8;101] | 0.770 |

p.AKI and sepsis vs No-AKI and sepsis: NHE3: 0.097; ENaC: 0.611; ROMK: 0.263; AQP2: 1.000

p.AKI and ischemia vs No-AKI and ischemia: NHE3: 0.234; ENaC: 0.739; ROMK: 0.991; AQP2: 0.874

p.AKI and ischemia vs AKI and sepsis: NHE3: 0.956; ENaC: 0.611; ROMK: 0.215; AQP2: 0.874

p.No-AKI and ischemia vs No-AKI and sepsis: NHE3: 1.000; ENaC: 0.739; ROMK: 1.000; AQP2: 0.874

Supplemental Table 4: Characteristics of patients, by in-hospital death.

|  | **Non-survivors (N=12)** | **Survivors (N=40)** | **p-value** |
| --- | --- | --- | --- |
| Age, years, median [IQR] ^&^ | 63.0 [56.0;66.0] | 56.0 [39.8;65.8] | NS |
| Male sex, n (%)* | 0 | 21 ( 52.5) | 0.001 |
| White race, n (%) | 9 (75.0) | 31 (77.5) | NS |
| Body mass index, Kg/m^2^, median [IQR] ^&^ | 24.6 [23.0;27.6] | 22.6 [21.4;25.4] | NS |
| SAPS3 score, mean ± SD ^& *^ | 62.3 ± 13.6 | 46.4 ± 15.2 | 0.003 |
| SOFA score, median [IQR] ^# *^ | 5.50 [3.75;8.00] | 2.00 [0.75;4.00] | <0.001 |
| Non-renal SOFA score, median [IQR] ^# *^ | 5.50 [3.22;8.00] | 2.00 [0.52;3.00] | <0.001 |
| AKI risk prediction clinical score ^7^, median [IQR] ^&*^ | 7.50 [6.50;9.00] | 5.00 [5.00;7.00] | 0.003 |
| Norepinephrine dose, μg/kg/min, median [IQR] ^&*^ | 0.08 [0.00;0.16] | 0.00 [0.00;0.08] | 0.047 |
| Mechanical ventilation, n (%) ^&*^ | 11 (91.7) | 23 (57.5) | 0.039 |
| Baseline creatinine, mg/dl, median [IQR] | 0.54 [0.44;0.57] | 0.56 [0.52;0.75] | NS |
| Urine output, ml/kg/h, median [IQR] ^#^ | 0.80 [0.68;1.02] | 0.88 [0.69;1.21] | NS |
| Fluid balance, ml/24h, median [IQR] ^#^ | 107 [-251.75;364] | 338 [-435.00;723] | NS |
| Serum creatinine, mg/dl, median [IQR]  ^#^ | 0.62 [0.57;0.70] | 0.64 [0.57;0.90] | NS |
| Serum urea, mg/dl, median [IQR] ^# *^ | 39.0 [35.2;48.2] | 22.5 [18.0;30.0] | 0.001 |
| **Supplemental Table 4 (continuation)** | **Non-survivors (N=12)** | **Survivors (N=40)** | **p-value** |
| Serum sodium, mEq/l, median [IQR] ^# *^ | 145 [142;149] | 141 [139;143] | 0.035 |
| Serum potassium, mEq/l, mean ± SD ^#^ | 3.66 ± 0.48 | 3.96 ± 0.42 | NS |
| Serum osmolality, mOsm/Kg, mean ± SD ^#^ | 297 ± 15.6 | 288 ± 10.0 | NS |
| Serum urea / serum creatinine ratio, median [IQR] ^# *^ | 57.7 [49.3;90.4] | 34.3 [28.2;46.0] | <0.001 |
| Urinary creatinine, g/l, median [IQR] ^#^ | 0.90 [0.76;1.10] | 1.10 [0.75;1.52] | NS |
| Urinary urea, g/l, median [IQR] ^#^ | 16.2 [9.78;18.1] | 12.1 [9.38;16.0] | NS |
| Urinary sodium, mEq/l, median [IQR] ^# *^ | 41.1 [22.7;63.1] | 105 [55.9;146] | 0.002 |
| Urinary potassium, mEq/L, mean ± SD ^#^ | 49.7 ± 22.7 | 52.5 ± 28.2 | NS |
| Measured urinary osmolality, mOsm/Kg H_2_O, mean ± SD ^#^ | 531 ± 200 | 625 ± 257 | NS |
| Estimated urinary osmolarity, mmol/l, mean ± SD ^#^ | 442 ± 144 | 543 ± 186 | NS |
| Urinary urea excretion, g/24h, median [IQR] ^#^ | 16.6 [13.9;27.9] | 17.6 [10.9;24.4] | NS |
| Fractional excretion of urea, %, mean ± SD ^# *^ | 26.0 ± 10.7 | 34.4 ± 12.5 | 0.032 |
| Urinary urea / urinary creatinine ratio, median [IQR] ^# *^ | 17.2 [12.2;21.5] | 11.2 [8.67;15.1] | 0.016 |
| Urinary sodium excretion, mEq/24h, median [IQR] ^# *^ | 45.5 [25.6;103] | 126 [65.1;243] | 0.004 |
| **Supplemental Table 4 (continuation)** | **Non-survivors (N=12)** | **Survivors (N=40)** | **p-value** |
| Fractional excretion of sodium, %, median [IQR] ^# *^ | 0.20 [0.10;0.32] | 0.41 [0.23;0.84] | 0.012 |
| Urinary sodium / urinary creatinine ratio, median [IQR] ^# *^ | 46.4 [26.7;88.6] | 88.6 [50.4;160] | 0.016 |
| Fractional excretion of potassium, %, median [IQR] ^#^ | 9.12 [6.30;10.4] | 7.77 [5.43;10.9] | NS |
| Urinary sodium+urinary potassium, mEq/l, median [IQR] ^# *^ | 103 [79.6;109] | 152 [121;189] | 0.001 |
| Diuretic use, n (%) | 1 ( 8.3) | 2 ( 5.0) | NS |
| Vasopressin use, n (%) | 2 (16.7) | 0 | NS |
| RAAS inhibitors use, n (%) | 1 (9.1) | 6 (15.0) | NS |

^#^ Data from ‘AKI’ group regards the day before AKI diagnosis. Data from ‘No AKI’ group regards the first five days average for each variable.

^&^ Data from the first day of ICU admission

NS: nonsignificant (p≥0.05)

Serum osmolality was estimated as: 2X sNa + sU/6 + sGlu/18, where sNa is serum sodium (in mmol/l), sU is serum urea (in mg/dl) and sGlu is serum glucose (in mg/dl)

Estimated urinary osmolarity was done as (uNa+uK) X 2 + uU, where uNa is urinary sodium, uK is urinary potassium and uU is urinary urea (solutes in mmol/l) ^13^

AKI: Acute Kidney Injury; IQR: interquartile range; n: number of patients; RAAS: renin-angiotensin-aldosterone system; SAPS: Simplified Acute Physiology Score; SD: standard deviation; SOFA: Sequential Organ Failure Assessment.

Supplemental Table 5: Multiple logistic regression model developed to check association of clinical variables with in-hospital mortality as the outcome.

|  | OR | CI 95% | p-value |
| --- | --- | --- | --- |
| SOFA | 4.50 | (1.75; 28.72) | 0.0257 * |
| Sepsis | 0.00 | (0.00; 0.34) | 0.0528 |
| Ischemia | 0.02 | (0.00; 0.99) | 0.1133 |
| Age | 1.21 | (1.05; 1.57) | 0.0484 * |
| uNa+uK | 0.97 | (0.93; 0.99) | 0.0748 |
| sNa | 1.32 | (1.06; 1.91) | 0.0482 * |

*AIC 32.24 and c-statistics: 0.97*

CI: confidence interval; OR: odds ratio; sNa: serum sodium; SOFA: Sequential Organ Failure Assessment; uK: urinary potassium; uNa: urinary sodium.
